# Commentary: Early Clinical Indicators of Addison's Disease in Adults With Type 1 Diabetes: A Nationwide, Observational, Cohort Study

**DOI:** 10.3389/fendo.2019.00456

**Published:** 2019-07-09

**Authors:** Jan Brož, Jana Urbanová, Katarina Halčiaková, Marisa A. Nunes, Ludmila Brunerová

**Affiliations:** ^1^Department of Internal Medicine, Second Faculty of Medicine, University Hospital Motol Charles University, Prague, Czechia; ^2^Center for Research of Diabetes, Metabolism and Nutrition, Prague, Czechia; ^3^Second Department of Internal Medicine, Third Faculty of Medicine, University Hospital Královské Vinohrady, Prague, Czechia

**Keywords:** type 1 diabetes mellitus, Addison's disease, screening, diabetic complications, severe hypoglycaemia

We read with great interest the article by Chantzichristos et al. (1) published in the *Journal of Clinical Endocrinology and Metabolism*. The paper presents the results of an observational, matched-cohort study which set out to determine whether there are any early clinical indicators for the onset of Addison's disease (AD) in adults with type 1 diabetes mellitus (T1DM). In the first part of the study, the authors analyzed many variables within a period of 2 years before diagnosis of AD in 66 T1DM patients, using data from several registries. Comparisons were made with 330 controls, matched for age, sex, and duration of diabetes. It was concluded that the presence of medical treatment for thyroid disease, a severe infection (expressed as necessity of hospital admission), and glucagon prescription for severe hypoglycemia should raise the suspicion of AD development in adults with T1DM. A significantly higher prevalence of diabetic retinopathy was also found in T1DM patients with AD.

The authors should be congratulated on their efforts to collect and analyse such a large amount of data and trying to correct for important covariables, leading to an interesting debate and highlighting this very important topic.

Although we agree with many of the conclusions of this interesting and important study, we would like to make at least four comments.

First, we understand that the authors consider the glucagon prescription as a sort of sign of severe hypoglycemia. However, it should be noted that this is only its indirect marker (2). Furthermore, although severe hypoglycemia was occasionally reported as a symptom of AD in TIDM patients (3–5), the study which aimed to screen for AD in patients with recurrent severe hypoglycemia failed to demonstrate the value of this as a screening test for the disease (6). Therefore, we would like to respectfully suggest investigating whether two or all three features mentioned in the conclusion are not clustering together in any of the patients in the AD group and compare it with those in the control group, as this could reveal another and probably more powerful marker of AD.

Second, it would be interesting to see not only one value of HbA1c but its full course during those 2 years before AD diagnosis. Its analysis may discover a possible impairment and thus find another marker of AD.

Third, although the prescriptions of various drugs were analyzed insulin was not involved. As a reduction in insulin dose was described as one of the possible signs of AD (7), analysis of the change in dose could reveal other important results.

Fourth, the prevalence of retinopathy (22.7%) in the AD group with even smaller number in the control group (13.6%) is much lower than that which was found, for example, in a Swedish population-based cross-sectional study (40%) conducted in a study population with similar mean diabetes duration (8). This difference may lead us to speculate whether the data in the registry reflect the real prevalence of diabetic retinopathy in the T1DM population.

We respectfully suggest taking these points into account especially if a continuation of this important study is planned.

## Author Contributions

JB designed and wrote the commentary. JU, KH, MN, and LB contributed to the design and revised the text critically for important intellectual content.

### Conflict of Interest Statement

The authors declare that the research was conducted in the absence of any commercial or financial relationships that could be construed as a potential conflict of interest.
